# Learning the Optimal Control of Coordinated Eye and Head Movements

**DOI:** 10.1371/journal.pcbi.1002253

**Published:** 2011-11-03

**Authors:** Sohrab Saeb, Cornelius Weber, Jochen Triesch

**Affiliations:** 1 Frankfurt Institute for Advanced Studies (FIAS), Goethe University Frankfurt, Frankfurt am Main, Germany; 2 Faculty of Mathematics, Informatics and Natural Sciences, University of Hamburg, Hamburg, Germany; University College London, United Kingdom

## Abstract

Various optimality principles have been proposed to explain the characteristics of coordinated eye and head movements during visual orienting behavior. At the same time, researchers have suggested several neural models to underly the generation of saccades, but these do not include online learning as a mechanism of optimization. Here, we suggest an open-loop neural controller with a local adaptation mechanism that minimizes a proposed cost function. Simulations show that the characteristics of coordinated eye and head movements generated by this model match the experimental data in many aspects, including the relationship between amplitude, duration and peak velocity in head-restrained and the relative contribution of eye and head to the total gaze shift in head-free conditions. Our model is a first step towards bringing together an optimality principle and an incremental local learning mechanism into a unified control scheme for coordinated eye and head movements.

## Author Summary

Human beings and many other species redirect their gaze towards targets of interest through rapid gaze shifts known as saccades. These are made approximately three to four times every second, and larger saccades result from fast and concurrent movement of the animal's eyes and head. Experimental studies have revealed that during saccades, the motor system follows certain principles such as respecting a specific relationship between the relative contribution of eye and head motor systems to total gaze shift. Various researchers have hypothesized that these principles are implications of some optimality criteria in the brain, but it remains unclear how the brain can learn such an optimal behavior. We propose a new model that uses a plausible learning mechanism to satisfy an optimality criterion. We show that after learning, the model is able to reproduce motor behavior with biologically plausible properties. In addition, it predicts the nature of the learning signals. Further experimental research is necessary to test the validity of our model.

## Introduction

Active perception of the visual world necessitates frequent redirection of our gaze. Such visual orientation behavior comprises multi-segment control of different motor systems, i.e. the coordinated movement of several parts of the body including the eyes, the head, and the torso. The coordinated movements of the eyes and the head during fast gaze shifts are called saccadic eye and head movements and are usually investigated in two conditions: head-restrained and head-free.

In the head-restrained condition, head movement is limited so that the gaze shifts rely only on eye movements. These eye movements, known also as eye-only saccades, possess certain physical properties. The relationship between the duration, peak velocity and the amplitude of saccades is known as the *main sequence* [1]. This relationship is stereotyped: the duration increases linearly with the saccadic amplitude, while the peak velocity increases linearly for low amplitudes and undergoes a soft saturation for larger amplitudes [2]–[4]. The velocity profiles of saccadic eye movements are smooth and symmetric for small amplitudes, while they become skewed for larger amplitudes [5], [6].

In the head-free condition, the head is allowed to accompany the eye in visual orienting. These movements are usually composed of two phases: in the first phase, the gaze is rapidly shifted to the target using both the eyes and the head. Once the gaze reaches the target, the second phase starts. In the second phase, the head continues moving in the same direction as in the first phase, but the eyes move backwards with the same velocity as the head. As a result, the gaze remains stabilized on the target. The general belief is that the vestibulo-ocular reflex (VOR) has a fundamental role in generating the coordination of eye and head during the second phase (see [7] for a review).

When the head is free to move, the kinematic characteristics of saccadic eye movements change dramatically compared to the head-restrained condition. As the gaze shift amplitude increases, the eye movement amplitude approaches its limits, and the head contribution becomes more prominent. Therefore, the eye's position and velocity is not determined only based on the current gaze error, but it also depends on concurrent head position and velocity. Furthermore, the eye's peak velocity declines in the head-free condition, its duration increases and its velocity profiles change [8]–[14].

### Previous Studies

Previous computational studies on saccadic eye and head movements revolve around two questions. The first question concerns the *optimality principles* underlying the kinematic characteristics, and the second one is about the *neural architecture* that generates appropriate control signals for driving eye and head muscles. Researchers dealing with both questions consider linear eye and head plants, which are equivalent to linear differential equations describing the mechanical properties of eye and head motor systems. Such models are considered sufficient for modeling the oculomotor and the head motor system dynamics [15], [16].

#### Optimality principles

During saccadic eye and head movements, visual information is not properly transmitted to the brain either due to motion blur or because of neural suppression induced by higher regions [17]. Therefore, saccadic gaze shifts should be as fast as possible in order to increase the amount of time the image is stabilized on the retina. This has been a fundamental assumption of many studies that were aiming at finding the optimality principles underlying the kinematic characteristics of saccades.

Early studies proposed that the saccade trajectories are optimized in such a way that they minimize the time to reach the target [18]. This assumption, known as the *minimum-time* principle, leads to a bang-bang control solution [19], for which the resulting velocity profiles are not biologically plausible [20]. Therefore, additional assumptions are necessary.

A key assumption suggested by Harris and Wolpert was that there exists additive white noise in the neural command, whose instantaneous power (variance) is proportional to that of the command signal [21]. Due to this assumption, the variance of the final eye position increases as one tries to decrease the saccadic duration by recruiting larger command signals. Therefore, in addition to the saccadic duration, the variance of the eye position should also be minimized. Because of this property, this principle is also called the *minimum-variance* principle. As a result of these two assumptions, a trade-off emerges between the speed and the accuracy of saccades, and the optimal solution to this trade-off is a trajectory that is biologically realistic [22].

Kardamakis and Moschovakis suggested another optimality principle based on the *minimum-effort* rule and optimal control theory [23]. This principle was used to obtain optimal control signals for both oculomotor and head motor systems in coordinated eye and head movements. The minimum-effort rule obliges that the squared sum of the eye and the head torque signals integrated over the movement period be minimized in order to obtain the optimal control signal. The optimization process uses boundary conditions for the gaze position and is only applied to the first phase of coordinated eye and head movements. In the head-restrained condition, this method achieves unimodal velocity profiles with shorter acceleration and longer deceleration phases compatible with many experimental findings [5], [6]. For the head-free condition, the contribution of eye and head to total gaze shift obtained by this method is biologically realistic, and the eye-velocity profiles become double-peaked as in the experiments [24].

#### Architectures

The functional architectures suggested for the control of saccadic eye and head movements can be categorized into two groups: architectures for head-restrained and head-free control problems. Since the goal of our study is to solve these two control problems using a single architecture, we review the existing models based on their structure and the optimization method they use rather than the control problem they are supposed to solve. From this perspective, models can be categorized into *feedback models*, which use gaze feedback to control saccades, and *independent control schemes* which do not need a gaze feedback.

The first gaze feedback model was suggested by Laurutis and Robinson [25] that was an extension of position control models of the saccadic system [26], [27] to incorporate gaze feedback signals. This model was thereafter used and extended by others [9], [28], [29]. The gaze error signal used in feedback models is internally estimated, such that no visual feedback is necessary. This is mainly due to two reasons. First, vision is impaired during fast gaze shifts, and second, there is a retinal processing delay of about 40–50 ms which can make the controller unstable [30]. Therefore, the gaze feedback signal can be regarded as an internal feedback. A more recent model by Chen-Harris and colleagues estimates the gaze feedback in a more elaborate way [31]. The internal feedback in this model consists of two forward models: a forward model of the oculomotor plant that predicts the state of the eye, and a forward model of the target motion that predicts the state of the target. This feedback used together with the absolute target position provides a signal to drive an optimized feedback controller which is based on the minimum-variance principle of Harris and Wolpert [22], and requires re-optimization for each saccadic duration.

The independent eye and head control models rely on the dynamics of their *burst generator* (BG) units rather than a gaze feedback in order to generate the control signals [12], [32], [33]. These BG units are *per se* closed loop controllers that use efference copies of eye and head motor command signals. Although the eye and the head control circuits have independent dynamics, there are ways through which they can influence each other. Independent control models usually assume that the relative contribution of eye and head components to the gaze shift is known beforehand. However, a recent neural model suggested by Kardamakis and colleagues [33] is able to reproduce realistic contribution of eye and head using the communication between the two circuits, and without such an assumption. The parameters of this model are either set according to experimental findings or optimized using a genetic algorithm.

### Our Contribution

The optimality principle studies by Harris and Wolpert [21] and Kardamakis and Moschovakis [23] have not provided any incremental learning mechanism for their optimization procedure. In fact, the optimization procedures used in these studies are based on Pontryagin's extremum principle [34], which requires boundary conditions at the initial and the final time of the saccadic movement, and provides a global analytical solution rather than a local adaptation mechanism. In the model suggested by Kardamakis and Moschovakis, the cost was evaluated for several values of gaze shift duration and the model parameters were eventually set to satisfy the trade-off between the effort and the duration. It may be speculated that such a solution can be a result of evolution, nevertheless, numerous experimental results indicate that saccadic eye and head movements are constantly adapted [35]–[37].

The neural control architectures that have been proposed to generate the eye and the head control signals do not use any optimality criteria to tune their parameters. The parameters of such models are usually hand-tuned or adjusted by a global optimization algorithm in a way that the model's response fits to the experimental data. The only exception we found is the model by Chen-Herris and colleagues [31] that relies on its internal feedback process to generate neural command signals.

As an alternative control scheme, we introduce an open-loop neural architecture. We try to obtain an adaptation mechanism that on the one hand can be implemented by the brain circuitry (see Discussion), and on the other hand minimizes a cost function. To this end, we suggest a cost function that does not directly depend on the saccadic duration, and therefore allows for a gradient descent based solution without any need to define boundary conditions. The control pathway of our model is feedforward, and is constantly calibrated by an adaptation mechanism that implicitly evaluates the optimality of the controller with respect to the cost function and induces parameter changes via a local learning rule. Therefore, our model can be regarded as a first step towards bringing together an optimality principle and an incremental local learning mechanism into a unified control scheme. It extends our previous model of eye-only saccade generation [38] to coordinated eye and head movements.

## Methods

**Figure 1:**
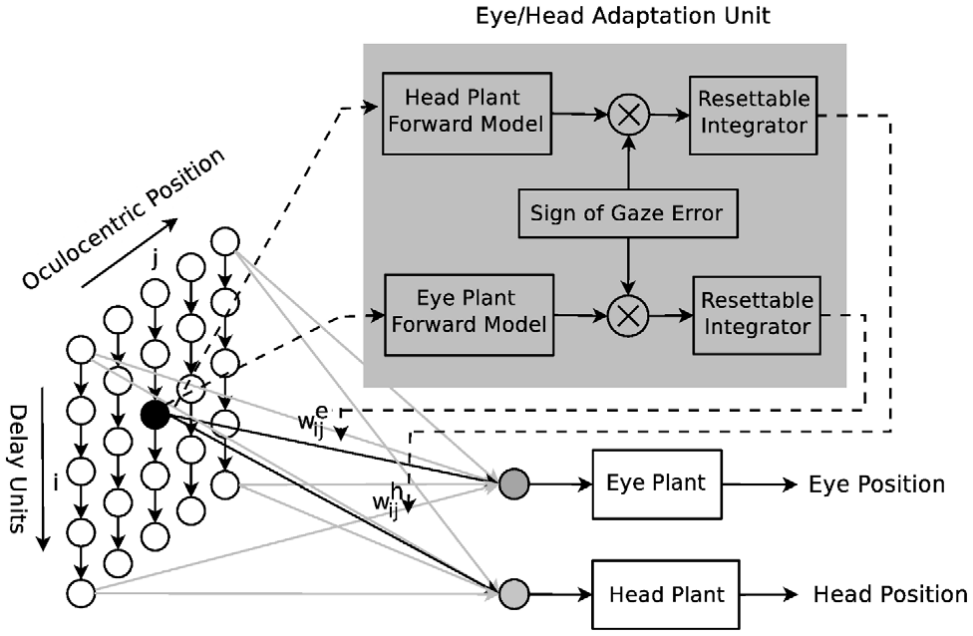
Model Architecture. The input, left, consists of one column of delay units per oculocentric position of the target. The read-out neurons (gray units; one for eye and one for head control) are linear and each weight parameter 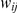 is adapted locally by the corresponding adaptation unit. The solid lines indicate the control signal pathway and the dashed lines represent the adaptation signal pathway.

The model architecture consists of two pathways: *feedforward control* and *adaptation*. The feedforward control pathway comprises a *spatiotemporal map* that performs spatial-to-temporal transformation as shown in Figure 1. The adaptation pathway is based on the learning rules derived from a cost function (see Adaptation).

### Spatial-to-Temporal Transformation

Saccades are produced by a precisely timed pattern of activity within the motor neurons innervating the eye and the head muscle systems. However, the desired gaze shift is represented spatially in areas such as the superior colliculus [39], [40]. This is called the spatial-to-temporal transformation problem (STTP) [41].

Here, we suggest a spatiotemporal map to perform such a transformation. This map comprises several columns (delay lines), each one including a number of neurons as shown in Figure 1. There is one column per oculocentric position of the target, i.e. the desired gaze shift amplitude. Only one visual dimension (e.g. the horizontal position) is modeled. The activity of the neurons in the columns is only dependent on the desired gaze shift amplitude and the progress of time. The spatial-to-temporal transformation is accomplished when these activities are integrated by two *read-out neurons* (gray units in Figure 1) to create the neural control signals that drive the eye and the head plants.

When an object triggers the initiation of a saccade, a single column corresponding to the desired gaze shift amplitude is activated. The activation of a column means that a wave of activity propagates through the neurons of that column, starting from the first neuron. The firing rate of each neuron changes as a Gaussian function. Given column 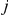 is activated at time 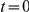, this propagation can be formulated as:
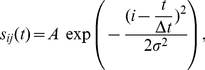
(1)where 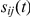 represents the instantaneous firing rate of the neuron 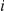 in column 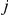, 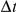 is the sampling period, 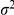 is the variance, and 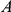 scales the height of the activity peak.

The two linear read-out neurons integrate the activity of the spatiotemporal map by means of weighted connections. This linear combination forms the neural command signals, 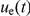 and 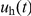, needed to drive the eye and head plants:
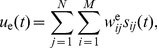
(2)

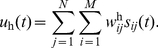
(3)



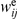
 and 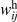 represent the weighted connections between neuron 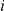 in column 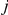 and the eye and head read-out neurons, respectively. 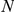 is the total number of columns and 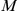 is the number of neurons in each column. Since we allow the neural command signals, 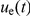 and 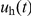, to become negative, we consider them as the difference between the firing rates of the agonist and the antagonist motoneurons [42] driving each plant.

The response of the eye plant is the eye position in head coordinates, 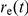, and the response of the head plant is the head position in body coordinates, 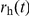. The details of these plant models as well as their corresponding responses are given in Text S1.

### Adaptation

The adaptation mechanism modifies the connection weights of the neural controller through several trials, such that it approaches an optimal behavior. Since the optimal behavior is determined by a cost function, adaptation implies the minimization of that cost function.

Before introducing the cost function, let us define the gaze error as:
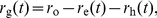
(4)where 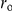 is the target object position in body coordinates, and 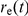 and 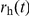 as defined before. For simplicity, we have assumed that the axes of eye and head rotation are perfectly aligned.

**Figure 2:**
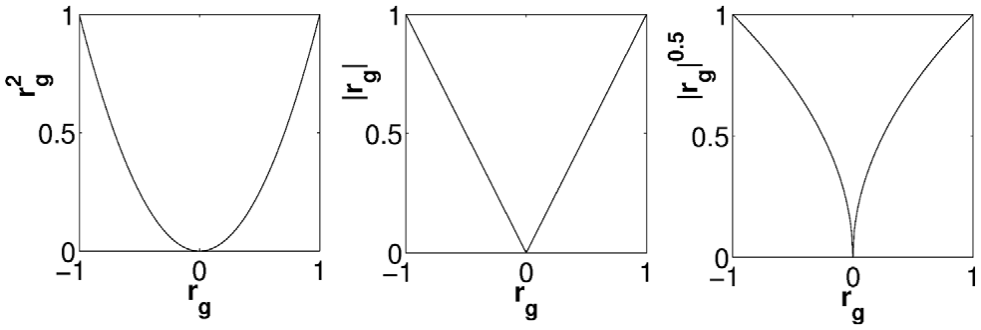
The first term of the cost function as a function of 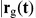. Three example functions (quadratic, absolute value and square root) are shown here.

We define a cost function that addresses the following objectives:

1. The gaze should reach the target as soon as possible and then stand still on the target position. Therefore, the cost function should depend on the absolute value of the gaze error, 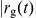. This dependency can be established via any arbitrary function of 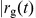, three examples shown in Figure 2. Convex functions such as a quadratic function do not seem a good choice since they do not penalize small gaze errors. We will proceed with the absolute value function because it results in more compatibility with neurophysiological observations, as we will see in the Discussion.
2. The power of the neural control signal should be constrained. This assumption may be viewed as a regularization [43]. It also addresses the problem of signal-dependent noise [21], as it reduces the variability of the neural control signal by preventing its power from becoming too large. Since the neural control signal is linearly dependent on the weight values, the cost function should depend on the absolute values of the weight parameters. Thus, large values of these parameters will be penalized regardless of their sign.

Accounting for these objectives, we formulate the cost function as:
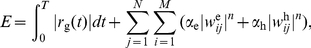
(5)


The time integral starts at saccade onset 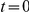, and 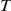 has a sufficiently large value so that the integral covers the whole movement duration. 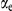 and 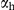 are positive coefficients determining the contribution of the eye and head weight limiting terms to the total cost, respectively. We set 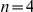 since this value leads to the results which have the most similarity to the experimental data.

It is worth noting that the integration time 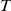 also covers part of the fixation period. This property of the proposed cost function facilitates the derivation of weight adaptation rules in case of delayed visual error, as studied on humans [44] and on macaque monkeys [45]. These studies show that a delayed visual error signal, up to several hundred milliseconds, is still able to induce saccadic adaptation.

The adaptable parameters of our model are the weights projecting from the spatiotemporal map to the two read-out neurons. We use a gradient descent method for minimizing the cost function. Using this method, the weight update rules are obtained as (see Text S1):
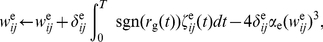
(6)

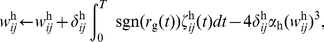
(7)where 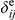 and 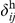 are adaptation rates, 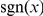 is the signum function, and:
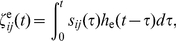
(8)

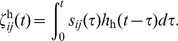
(9)


The functions 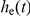 and 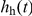 represent the impulse responses of eye and head plants, respectively.

The block diagram representation of the adaptation mechanism is shown in Figure 1 (gray area). This representation is inspired by Equations 6–9 in the following way: the signals 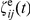 and 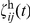 can be regarded as the responses of the *forward models* of the eye and the head plants, respectively. These forward models basically have the same impulse response as the eye and head plants while receiving a copy of the neural activity in the columns as input. The responses of these forward models are multiplied by the sign of the gaze error and then integrated over 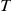 (see Equations 6 and 7). The resulting signals act on the same connection of the neuron that has stimulated the adaptation units. This influence is shown by a dashed arrow in Figure 1.

## Results

We consider two conditions: the head-restrained condition, where we set the head plant gain 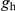 (see Text S1) to zero; and the head-free condition where we set 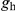 to its normal value, 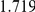. In biology, it is hypothesized that a neural gate prevents a common gaze shift command from reaching the neck circuitry when head-restrained saccades are desired [46].

For each condition, the learning procedure continues until the model reached a stable response. The simulation time step was 1 ms and 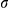 was set to 0.002.

We used 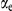 and 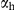 as the free parameters of our model to find the best match between the model behavior and experimental data. To this end, we used a genetic algorithm (GA) as described in Text S1. For the head-restrained condition, the GA fitness function was defined as the sum of squared errors (SSE) between the main sequence plots of the model and of the experiments [4]. The highest fitness value was found for 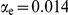. One should note that the value of 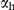 has no effect in the head-restrained condition since 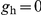 in this case. For the head-free condition, the fitness function was set as the SSE between the relative eye/head contribution of the simulated and of the experimental results, with eye position initialized at zero. The best parameters found in this case were 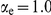 and 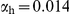.

### Head-Restrained Condition

**Figure 3:**
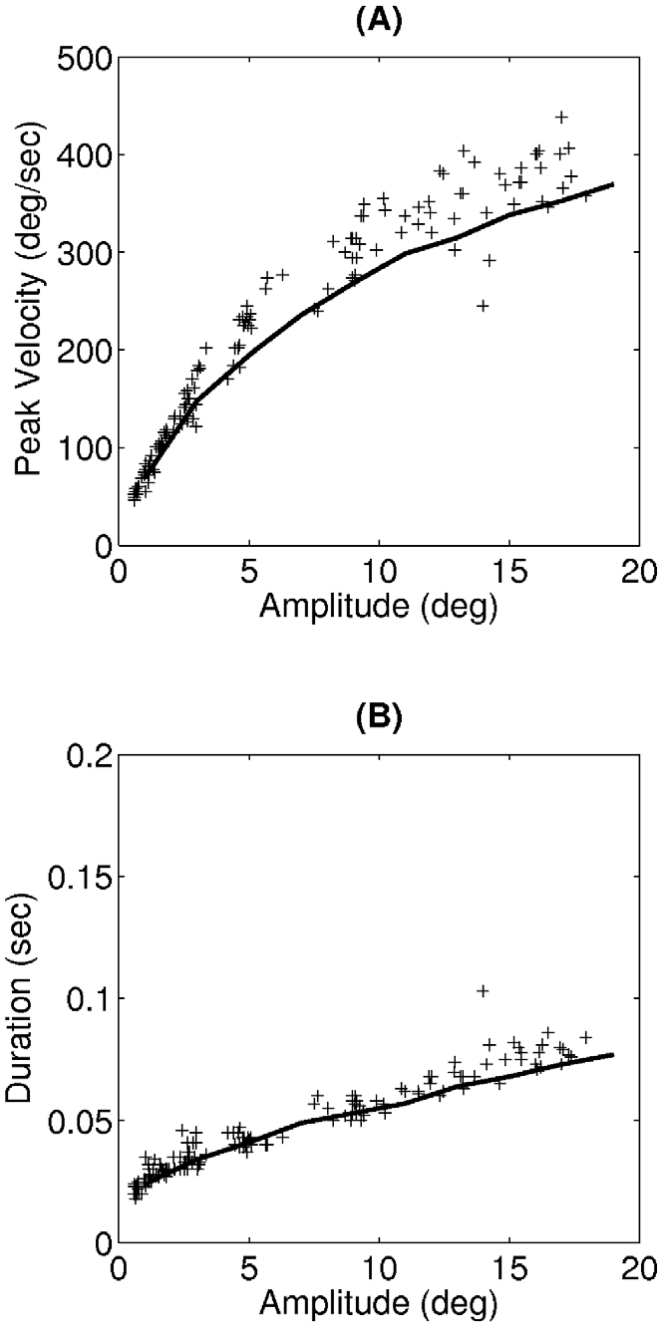
Comparing the main sequence plots of the proposed model to experimental data, in head-restrained condition. (A) Peak velocity and (B) duration of saccades versus their amplitudes. The solid lines represent the model results after learning, and the crosses are experimental data taken from an experiment on human subjects [4].

With the best model parameters found by the GA, we simulated the learning procedure (Equation 6) for different target object positions. The integration time was set to 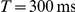, which was enough for learning saccadic eye movements for all amplitudes. We compared the simulation results to the experimental data obtained by Harwood and colleagues on human subjects performing horizontal eye movements [4]. This comparison was made between the main sequence plots, as shown in Figure 3.

**Figure 4:**
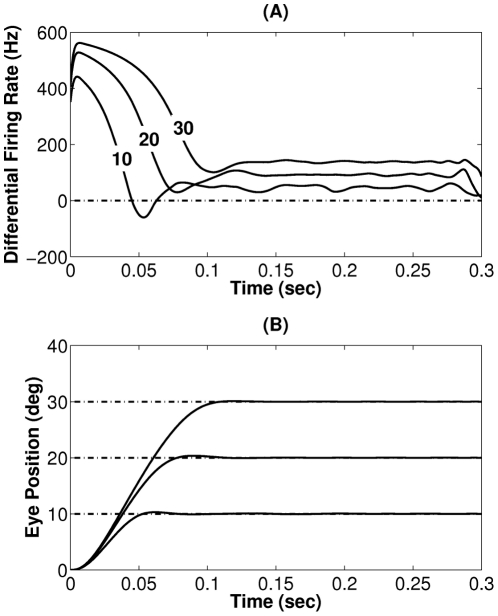
Model behavior after learning for saccades to targets at 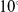, 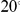, and 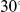 in head-restrained condition. (A) Optimized neural command signals of the eye defined as the difference between agonist and antagonist neural commands. (B) Eye position (eccentricity) in head coordinates. Target positions are shown by dashed lines.

The resulting neural control signals and their corresponding plant responses for three target object positions, 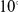, 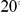, and 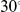, are depicted in Figure 4. These signals comprise two main phases: the saccadic phase during which the control signal is strong; and the fixation phase when it has a roughly constant but slightly oscillating positive value. The mean value of the neural control signal in the fixation period is proportional to the target position, and the small oscillations lead to slight eye drifts that are negligible because of their low contribution to the cost function. In fact, the eye plant filters out the high frequency inputs so that the eyes do not follow these oscillations. The decrease of the firing rate at the end of the plot is a boundary effect. No matter how long the integration time 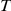 is, this effect is always observed at the final time.

**Figure 5:**
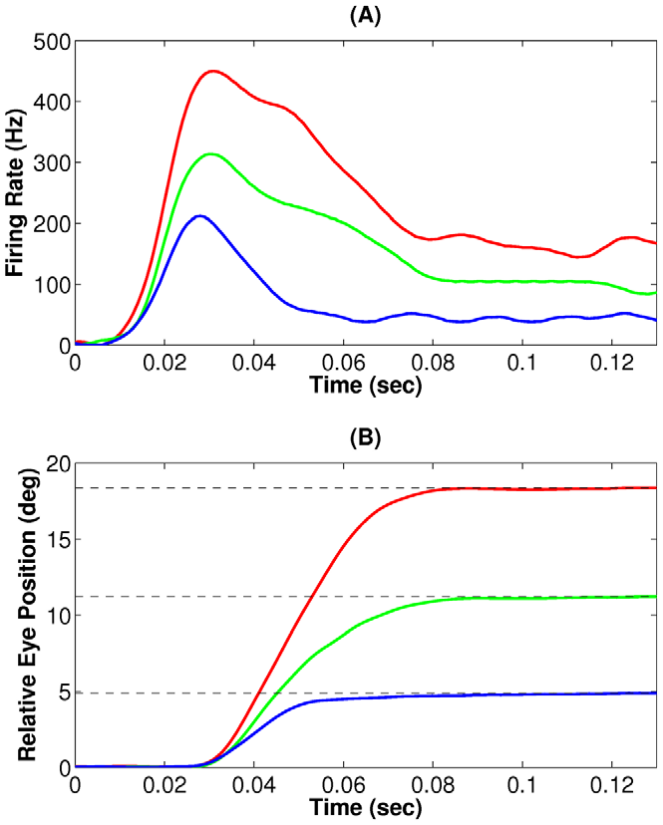
Experimental data from concurrent recording of motor neurons activities and eye position during head-restrained gaze shifts. (A) Firing pattern of an abducens nucleus (ABN) motor neuron during saccades with different amplitudes, coded by different colors. (B) The resulting change in the eye position. Both neural activity and eye position signals are vertically shifted such that they have zero initial values. Dashed lines show target (final) eye positions. Data are obtained from an experiment on rhesus monkeys [70], and are provided by M. Van Horn and K. Cullen.

The general form of the optimized neural control signals shown in Figure 4 resembles the firing patterns of abducens nucleus motoneurons in monkey responsible for saccadic eye movements, shown in Figure 5: a fast increase in the firing rate is followed by a slow decrease (the *burst* phase), then follows an oscillatory steady state that maintains the fixation (the *tonic* phase). During fixation, in both model and experimental data, the sustained tonic firing rate is proportional to the eye position. However, one should note that the firing rate patterns shown in Figure 4 are differential, i.e. they are obtained as the difference between the activity of agonist and antagonist motor neurons, while the ones shown in Figure 5 are not.

**Figure 6:**
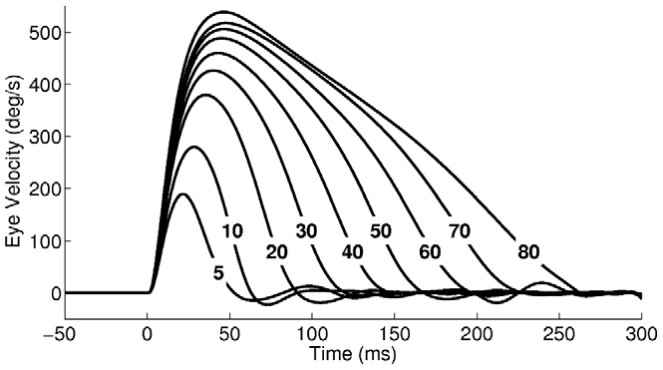
Adapted eye velocity profiles during the head-restrained condition for target positions from 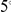 to 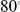. For comparison to experimental results, see for example Figure 2 of [6].

Without changing the model parameters we tested if the model is capable of reproducing realistic velocity profiles. For this, we simulated the learning process for the amplitudes ranging from 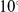 to 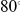. The velocity profiles corresponding to different saccadic amplitudes are shown in Figure 6. For small amplitudes, the profiles are smooth and almost symmetric, while for larger amplitudes they become skewed. The main reason for the former symmetry is that the effect of weight updating mechanisms (Equations 6 and 7) on the saccadic velocity is symmetric when the second term of the cost function (Equation 5) is small enough. This effect becomes biased against large weights when the second weight regularization term grows as a result of an increase in target eccentricity. The same trend is observed in experimental results, an example is presented in a study by Collewijn and colleagues [6] (see Figure 2 of this paper). It is worth noting that to make these large eye-only saccades possible in such experiments, for each saccadic amplitude *A* the saccade is made from −*A*/2 to +*A*/2 relative to the central fixation point on the horizontal meridian. For instance, a 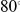 saccade is made by moving the eyes from 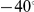 to 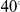 in head coordinates.

### Head-Free Condition

For the head-free condition, we again used the parameter values obtained by the GA and compared our results to experimental data from a study on rhesus monkeys [10]. The integration time 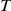 was set to 2 seconds for allowing the model to learn slow head movements. We let the model learn the gaze shifts for object positions ranging from 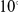 to 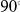 with a step size of 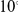, and for different initial eye positions.

**Figure 7:**
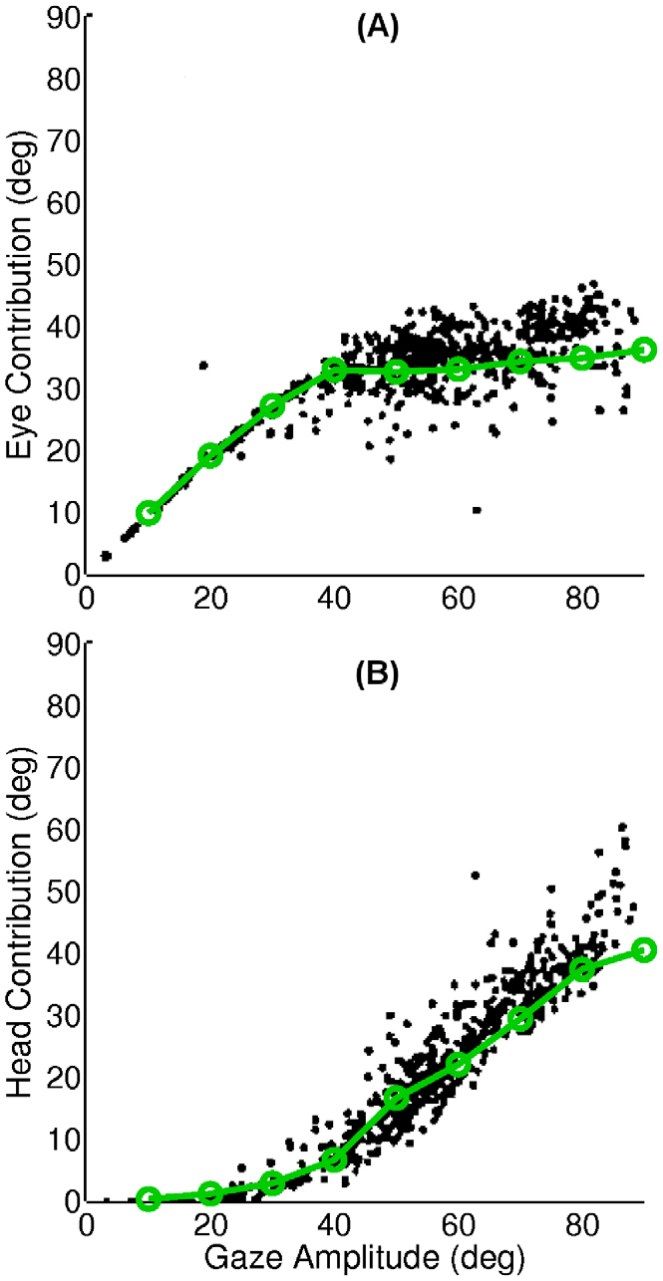
Relative contribution of eye and head to total gaze shift for different gaze shift amplitudes. (A) Eye contribution calculated as the relative displacement of the eye from the beginning until the end of gaze shift. (B) Head contribution calculated in the same way. Dots are experimental data from a study on rhesus monkeys making horizontal gaze shifts [10]; and green circles are model simulation results.

**Figure 8:**
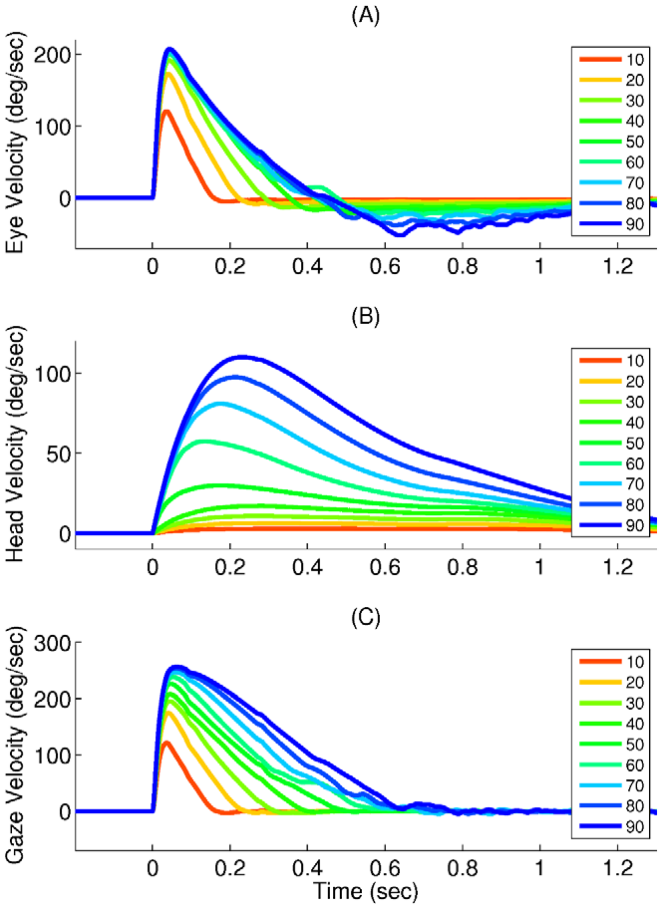
Velocity profiles generated by the proposed model, for different gaze shift amplitudes in head-free condition. (A) Eye, (B) head, and (C) gaze velocity profiles. The color codes for different gaze shift amplitudes in degrees (see the legends).

Experimental studies have revealed that the relative contribution of eye and head to total gaze shift varies depending on the gaze shift amplitude [10]. To see if our model is able to reproduce these observations, we have defined two quantities in compliance with the mentioned studies: first, the *eye contribution* to the gaze shift, which is defined as the amplitude of the eye movement that occurs between the eye movement onset and gaze movement end. Second, the *head contribution* to the gaze shift that indicates the head movement amplitude within this period. These two quantities are sketched in Figure 7 for object positions ranging from 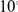 to 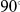 and initial eye position equal to zero. In both model and experimental data, the head contribution keeps increasing while the eye contribution undergoes a soft saturation as a function of gaze shift amplitude. This behavior is also evident in the eye and head velocity profiles in Figure 8. While the head peak velocity increases proportionally with the gaze shift amplitude, the eye peak velocity saturates for very large gaze shifts (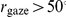).

**Figure 9:**
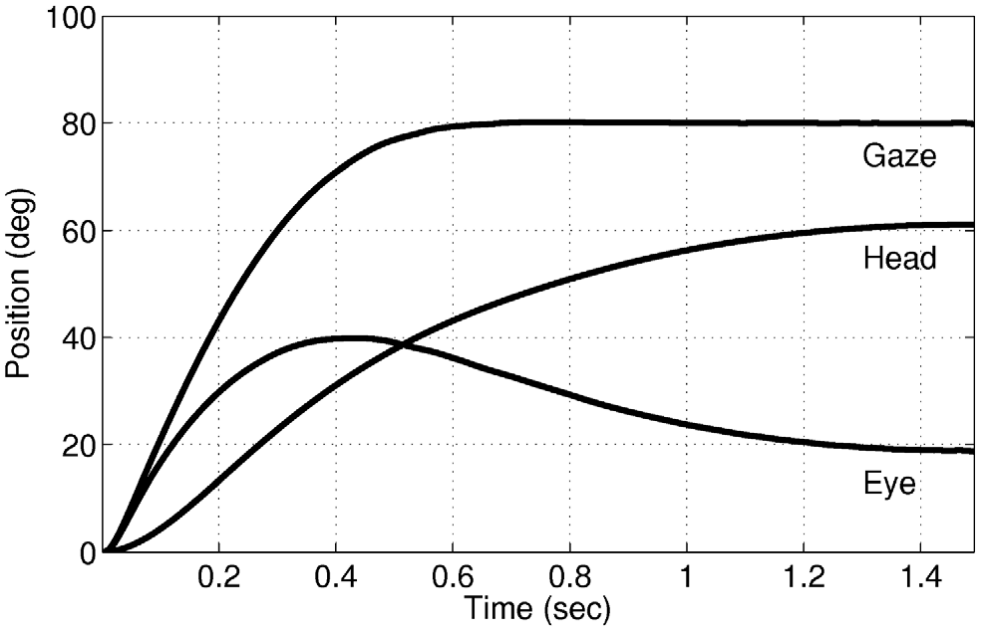
Eye, head and gaze positions for a 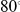 gaze shift. The two phases of coordinated eye and head movements, rapid gaze shift and VOR-like behavior, are evident.

The two phases of coordinated eye and head movements, i.e. the rapid gaze shift phase and the VOR-like behavior, are evident in the position plots shown in Figure 9. These two phases can also be observed in the eye velocity profiles (Figure 8A), where the eye velocity is positive during the first phase and negative during the second.

**Figure 10:**
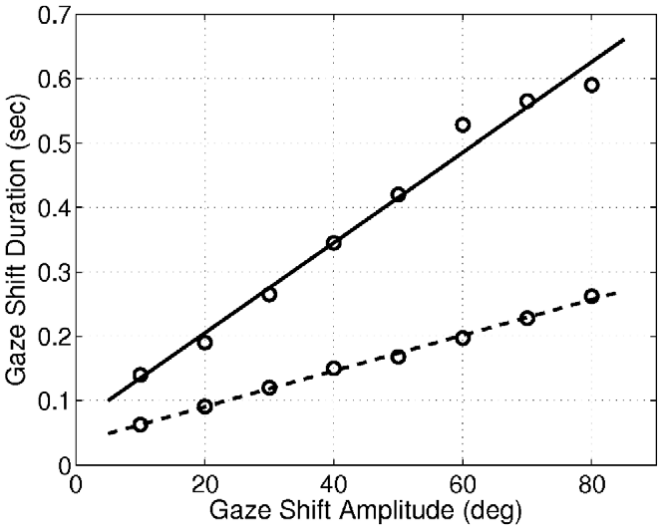
Gaze shift duration in the proposed model, for head-restrained compared to head-free conditions. The dashed line shows the head restrained and the solid line the head-free condition. The duration is in general higher in the head-free condition, and the difference increases by increasing the gaze shift amplitude. Circles show sampled data, and the lines are fitted by linear regression using the least squares approach. The correlation coefficients (*r*) and the slopes (*s*) are 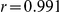 and 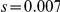 for the solid line and 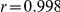 and 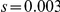 for the dashed line.

According to our model, the main reason for the observed increase of the head contribution compared to that of the eye is the existence of slower poles in the head plant that require more time to produce a considerable response. For very low gaze shift amplitudes (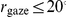) the eyes rapidly catch the target before the head plant accelerates; therefore the head contribution is almost zero. For larger gaze shift amplitudes, the head has enough time to accelerate since the eye plant saturates due to the cost on its neural command signal. Thus, the increase in the head contribution gradually dominates the increase of the eye contribution, leading to the results shown in Figure 7. Compared to the head-restrained condition, the gaze shift duration is longer for the same gaze shift amplitudes. This difference increases almost linearly by increasing the amplitude (Figure 10; also compare Figure 8C with Figure 6), which is compatible with experimental results [47].

**Figure 11:**
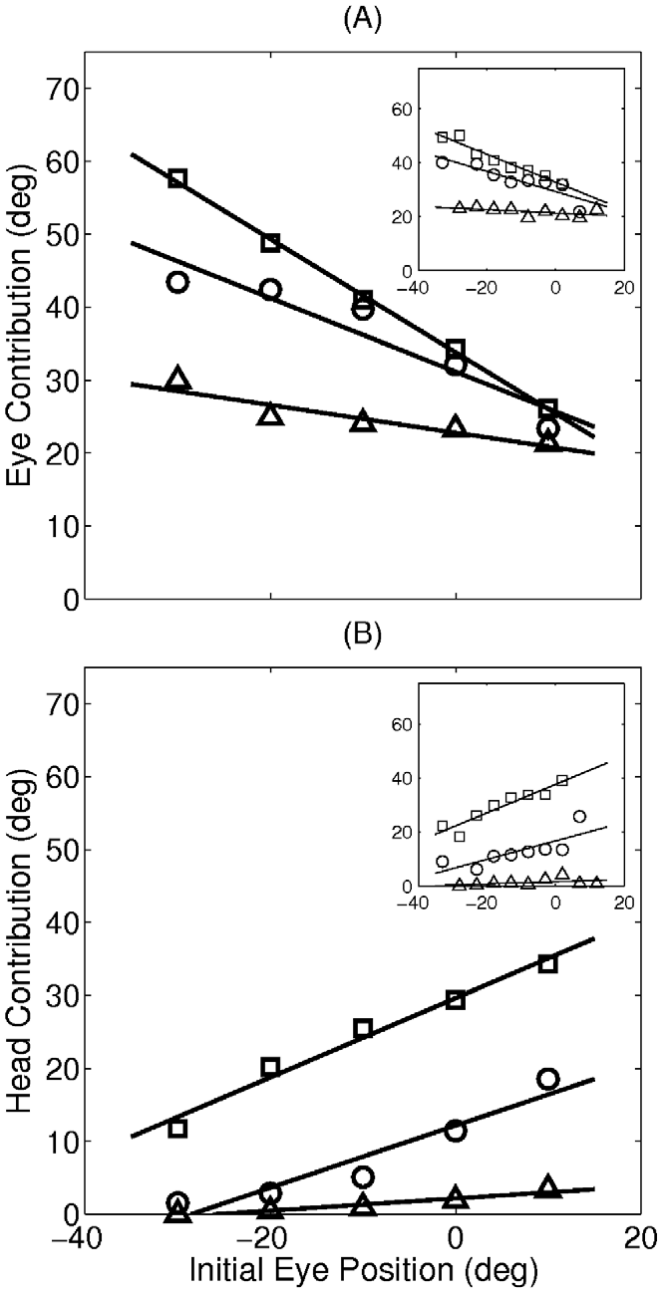
Eye and head contribution to the gaze shift as a function of initial eye position. (A) Eye and (B) head contribution obtained for the gaze shift amplitudes of 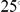 (triangles), 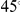 (circles) and 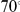 (squares). Main plots show model results after learning, and insets illustrate the mean value of experimental data extracted from a study on rhesus monkeys [10]. The linear fits conducted both on the model and on the experimental data are obtained by linear regression using the least squares approach. The correlation coefficient (*r*) and the slope (*s*) of each line is as follows. For the model data, Panel A: 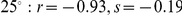; 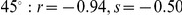; 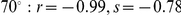. Panel B: 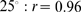; 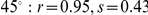; 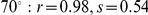. For the experimental data, Panel A: 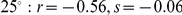; 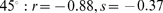; 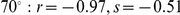. Panel B: 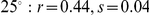; 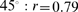; 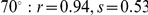.

Experimental studies have also shown that the relative contribution of eye and head to total gaze shift depends on the initial eye position in head coordinates [10], [29], [30], [48]. In fact, gaze shifts with identical amplitudes can be constructed of eye and head movements having a variety of amplitudes. To check for the ability of our model to reproduce this behavior with the same set of free parameter values, we ran a second set of simulations. In these simulations, three gaze shift amplitudes (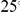, 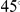, and 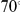) were learned for different initial eye positions (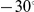, 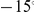, 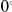 and 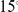). We compared the results of our simulation to experimental data obtained from a study on rhesus monkeys [10] in Figure 11. In both model and experimental results, when the eyes are initially deviated away from the movement direction (negative initial eye positions), the head contributes less - and consequently the eyes contribute more - compared to the situation where the initial eye position is deviated in the direction of the gaze shift (positive initial eye positions). In terms of our model, this behavior can be explained by looking at the neural command signals that are necessary in each situation: if we consider no contribution from the head, the final eye position will only depend on the initial eye position, such that the eye movements starting from more positive initial positions end up with higher final positions. This requires an overall larger neural command signal compared to negative initial eye positions, and according to the proposed cost function, larger command signals impose higher costs. To decrease this cost, the head should contribute more when the initial eye position is more positive.

## Discussion

Using the architecture shown in Figure 1 and considering a simple cost function defined by Equation 5, we were able to reproduce the fundamental characteristics of coordinated eye and head movements in both head-restrained and head-free conditions. The proposed optimality principle has some similarities, as well as differences, to existing principles [22], [23]. A point-by-point comparison between our model and other principles including the minimum-time, minimum-variance, and minimum-effort is given in Table 1.

**Table 1:** Comparing the proposed model to other models in various aspects.

Feature	Minimum-Time [18]	Minimum-Variance [21]	Minimum-Effort [23]	Our Model
Main Sequence	✓	✓	✓	✓
Realistic Velocity Profiles in Head-Fixed Condition	✗	✓	✓	✓
Eye Fixation in Head-Fixed Condition	✗	✓	✗	✓
Eye-Head Coordination	✗	✗	✓	✓
Neural Implementation	✗	✗	✓	✓
VOR-like behavior in Head-Free Condition	✗	✗	✗	✓
No Boundary Conditions	✗	✗	✗	✓
Incremental Learning	✗	✗	✗	✓
Generalized to other tasks	✓	✓	✗	✗
Double-Peaked Eye Velocity Profiles in Head-Free Condition	✗	✗	✓	✗

A substantial difference of the new cost function from previous ones is that it does not directly penalize the gaze shift duration. Instead, it punishes the total gaze error integrated over an arbitrary time interval (*T*) that is large enough to encompass the gaze shift period. This has two benefits: first, it allows for the application of the gradient descent method, since the total gaze error can be expressed directly in terms of an unknown neural command signal (see Text S1). This is not possible for the other principles due to the fact that there exists no closed-form expression of the gaze shift duration in terms of the neural command. The incorporation of gradient descent into optimization means that the optimization process turns into an incremental learning process, which can be regarded as a step forward in the direction of a biologically realistic implementation.

The second advantage of the arbitrary integration time in Equation 5 is that it also covers part of the post-saccadic response. This implies that our model is also able to generate the motor commands needed immediately after the gaze shift, whereas the previous models have only attempted to explain the gaze shift phase. In the head-restrained condition, this is just before the visual feedback from the target is re-established to keep the eye position still on the target. For the head-free condition, the model is able to reproduce a VOR-like behavior, where the eyes move back toward their central position in head and at the same time the head continues moving such that the gaze remains stabilized on the target.

As pointed out in Table 1, the proposed optimality principle, along with minimum-effort, is able to reproduce not only the main sequence behavior in the head-restrained condition, but also the coordination of eye and head during head-free gaze shifts, whereas minimum-variance is not. Nevertheless, the minimum-variance principle has been successfully generalized to other motor control tasks such as arm movements [21]. Apart from minimum-time, all of the models are able to generate biologically realistic velocity profiles for eye-only saccades, but only the minimum-effort model is capable of reproducing double-peaked eye-velocity profiles [23] that are observed experimentally during head-free gaze shifts [24].

In the simulation results, the value of 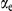 in the head-free condition (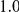) is considerably larger than in the head-restrained condition (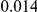). This implies that the resulting eye controller weights 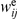 and consequently the average amplitude of the eye command signal 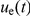 should be smaller in the head-free condition. This effect might be related to the hypothesis that the head velocity signal inhibits the gain of the saccadic BG units [12].

The learning mechanism introduced by Equations 6 and 7 necessitates the existence of eye and head internal models that provide 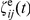 and 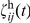. These forward models respond to the activity of individual neurons in the spatiotemporal map. In addition, since vision is impaired during saccades, there should exist another internal forward model which provides the sign of the gaze error to the adaptation mechanism (see the adaptation unit in Figure 1) using efference copies of the current neural control signals, 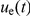 and 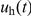, as input.

The cerebellum is widely regarded as a neural substrate where internal models of the motor system are located (see [49] for a review), and the most convincing neurophysiological data for internal models has been obtained for eye movements [50]. Bastian suggests that the cerebellum performs feedforward correction on the movement based on the error assigned to the previous movement [51]. Interestingly, an experimental study by Soetedjo and Fuchs indicates that the complex spike activity of Purkinje cells (P-cells) in the vermis of the oculomotor cerebellum signals the sign (direction) but not the magnitude of the gaze error during saccade adaptation [52], a finding which is consistent with the adaptation mechanisms of our model. Furthermore, several studies have revealed that cerebellar lesions permanently annihilate the adaptive capabilities of saccadic eye movements [36], [53]–[55], which suggest that the saccadic system is constantly calibrated by the cerebellum. Specifically, the study of Buettner and Straube showed that bilateral lesions in the cerebellar vermis lead to hypometric saccades [54]. This effect can be reproduced in our model by eliminating the adaptation signal (the first term in Equation 6). In such a situation, the weight decay term will decrease the weight values, leading to saccades that are smaller than the desired gaze shift.

Based on the mentioned studies about the cerebellum, we speculate that the adaptation signals affecting the feedforward controller are likely to be produced by the cerebellar vermis. This assumption, however, requires several parallel implementations of the eye and head forward models for each weighted connection in the feedforward control pathway. The existence of several parallel microzones in the cerebellum that receive inputs via different sets of mossy fibers and project their outputs via distinct P-cells [56] offers a possible neural basis for that, but further investigations on the exact functionality of these microzones are necessary.

So far we speculated on possible neural substrates responsible for the adaptation. Now we look for possible neural substrates that are maintaining the open-loop (feedforward) control of saccadic gaze shifts. Takemura and colleagues analyzed the relationship between the firing patterns of the P-cells in the ventral paraflocculus (VPFL) area of the cerebellum and the following ocular responses [57]. They used a second-order linear regression method to reconstruct these signals based on three aspects of the eye movement: position, velocity and acceleration. This second-order linear method was able to reproduce temporal firing patterns of VPFL neurons. When a single set of coefficients was used for different visual stimuli in order to reconstruct the firing pattern of the cells in the VPFL, the best fits were found for P-cells in this area. This observation implies that there is a linear relationship between the firing pattern of P-cells in the cerebellar VPFL and the eye kinematics. Hence, the cerebellar VPFL is a possible candidate for the neural controller of our model. More specifically, we can consider the neural delay line structure as a model of the granular layer and the read-out neuron as a P-cell, in accordance with the cerebellar models which assume the granular layer as a basis for the spatiotemporal representation of the input signals and the P-cell layer as a layer that receives weighted projections from the granular layer [58], [59].

Another possible candidate for the open-loop neural controller is the superior colliculus (SC). It has been assumed that the caudo-rostral spread of activation emerging among the build-up cells of the SC is caused by an internal feedback signal during saccadic eye movements [28], [60], [61]. One of the most important predictions of these models is that interrupting this spread should delay the arrival of the activity at the rostral SC, and the eye should reach the target with delay. However, a lesion experiment performed on the SC does not support this idea: Aizawa and Wurtz observed that instead of delaying the reach time, the lesion results in a curved trajectory that does not end at the target position [62]. Motivated by this observation, Nakahara and colleagues suggested a computational model of the SC in which the spread of activity is a mere epiphenomenon of the asymmetric connections within the SC [63]. This suggestion supports our assumption that the neural activity propagation in the delay lines is a self-reliant process which does not depend on any external feedback, and makes the SC a strong candidate for the spatiotemporal map in our model. Furthermore, experiments have revealed that within the projections from the SC to BG neurons, stronger connections are correlated with larger saccade amplitudes [64]. This supports the assumption that the spatiotemporal transformation in the saccadic systems relies on the SC-BG projections. Nevertheless, in a model of the saccade generating system suggested by Optican and Quaia, the neuronal activity does not spread on the SC. Instead, together with a saccade velocity feedback signal, it causes a wave of activity on the cerebellar fastigial nucleus (FOR) that drives the BG neurons [65], [66].

When comparing model simulation results to experimental data, the reader should note that part of the data is obtained from monkeys while the other part is captured from human subjects (see the figure captions). This discrepancy is primarily due to the fact that appropriate data were not available for either monkeys or human subjects in order to make precise comparisons between the model and the primates behavior. In fact, the monkeys gaze shift behavior is very similar to that of humans, and there are only slight differences which are due to different mechanical properties of the oculomotor system between the two species [67]. These differences, however, are negligible in our study as they do not have a severe impact on the proposed computational principles.

**Figure 12:**
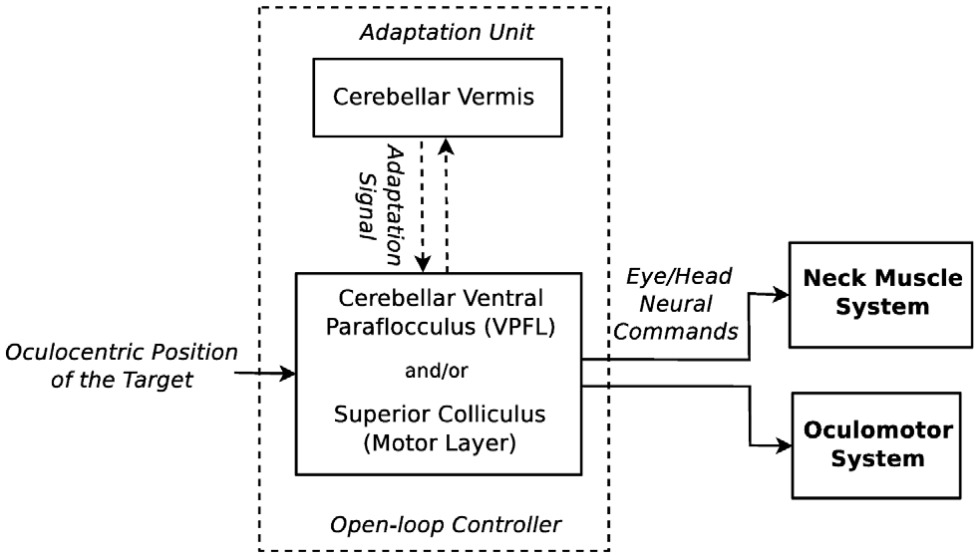
A possible simplified biological interpretation of the model architecture. The cerebellar VPFL and the motor layer of superior colliculus (SC) are candidates for the open-loop control of saccades while the cerebellar vermis is possibly responsible for providing the adaptation signals.

The aim of this study was to keep the proposed computational model as simple as possible, and to extend it only if there was some aspect which could not be addressed by the simple model. Therefore, the brainstem BGs and the motoneurons are not distinguished in our model, and the read-out neurons in Figure 1 are a simplified representation of the brainstem-motoneuron circuitry. Figure 12 illustrates a summary of our speculation on possible neural substrates responsible for the control and the learning of saccades. Indeed, more experimental investigations are needed to clarify the contribution of the cerebellum or the SC to the open-loop control of saccadic eye movements. As for adaptation, the signals produced by the adaptation unit of our model should be compared to signals that are transmitted from the cerebellar vermis to the brainstem when saccades are executed.

### Future Work

The present model only addresses the generation of saccadic gaze shifts along one spatial axis, requiring one column (delay line) for every target position along this axis. A naïve approach to generalize the model would be to introduce one column for every oculocentric position. However, this would require a very large quantity of neurons. As an alternative approach, one can introduce two separate 1-D controllers for the horizontal and vertical components of a gaze shift. Such an approach has been successfully implemented in [68]. Another open issue is the neural implementation of the forward models used by the neural controller, and a model that describes how the parameters of such forward models are adapted. To this end, one could use the temporal sequence learning approach [69] to perform forward model learning. Finally, the proposed open-loop controller can be generalized to involve other ballistic motor control tasks beyond coordinated eye and head movements, by finding appropriate cost functions that underly those tasks.

## Supporting Information

Text S1 Text S1 supplies detailed information on *Eye and Head Plant Models*, linear models of the eye and head dynamics used throughout this study; *Gradient Descent Optimization*, the optimization method used to derive the learning rules (Equations 6–9); *Adaptive Learning Rate Method*, a method for accelerating the learning procedure; and *Implementation*, describing the genetic algorithm method used for optimizing the free parameters of the proposed model.
(PDF)
