# Supplementary material for: Learning the Optimal Control of Coordinated Eye and Head Movements
Source: PLoS Comput Biol. 2011 Nov 3;7(11):e1002253. doi: 10.1371/journal.pcbi.1002253 (PMC3207939; doi:10.1371/journal.pcbi.1002253)
Supplement: Text S1 — supplies detailed information on Eye and Head Plant Models, linear models of the eye and head dynamics used throughout this study; Gradient Descent Optimization, the optimization method used to derive the learning rules (Equations 6–9); Adaptive Learning Rate Method, a method for accelerating the learning procedure; and Implementation, describing the genetic algorithm method used for optimizing the free parameters of the proposed model. (PDF) [file pcbi.1002253.s001.pdf]

Text S1: Supporting Information

1 Eye and Head Plant Models

The oculomotor and head motor systems can be modeled by linear pole-only plants. Previous studies have used 2- or 3-pole linear plants for the eye and 2-pole plants for the head [1–3]. Here, we consider a 3-pole plant for the eye, since the initial simulations of our control architecture showed that at least three poles are necessary to reproduce biologically-plausible velocity profiles in the head-restrained condition. Therefore, the impulse responses of the plants have the following general form:

$$h_{\mathrm{e}}(t) = g_{\mathrm{e}}(k_1 e^{-\frac{t}{T_1}} + k_2 e^{-\frac{t}{T_2}} + k_3 e^{-\frac{t}{T_3}}), \tag{1}$$

$$h_{\mathrm{h}}(t) = g_{\mathrm{h}}(k_4 e^{-\frac{t}{T_4}} + k_5 e^{-\frac{t}{T_5}}), \tag{2}$$

where T_x are the pole time constants, and g_{e} and g_{h} represent the DC gain values of the eye and head plants, respectively. For the head-restrained condition, since we are comparing our results to human experimental data, we set $T_1 = 224\,ms$, $T_2 = 13\,ms$, and $T_3 = 4\,ms$ as in a human eye plant model [4]. The head plant does not have any role in this condition. For the head-free condition, since we compare our results to monkey data, we set the parameters according to monkey eye and head plant models [4,5]. These values are $T_1 = 260\,ms$, $T_2 = 12\,ms$, $T_3 = 1\,ms$, $T_4 = 9844\,ms$, and $T_5 = 156\,ms$. The DC gain values are $g_{\mathrm{e}} = 0.217$ and $g_{\mathrm{h}} = 1.719$.

The coefficients k_1, k_2, ... k_5 can be calculated in terms of the pole time constants T_1, T_2, ... T_5 as:

$$k_1 = \frac{T_1}{T_1^2 + T_2T_3 - T_3T_1 - T_1T_2},$$
$$k_2 = \frac{T_2}{T_2^2 + T_3T_1 - T_1T_2 - T_2T_3},$$
$$k_3 = \frac{T_3}{T_3^2 + T_1T_2 - T_2T_3 - T_3T_1},$$

for the eye plant and:

$$k_4 = \frac{1}{T_4 - T_5},$$
$$k_5 = \frac{1}{T_5 - T_4},$$

for the head plant model. Therefore, each plant is parameterized by one DC gain and time constants of its poles.

The plant responses, $r_{\mathrm{e}}(t)$ and $r_{\mathrm{h}}(t)$, can be retrieved as:

$$r_{\mathrm{e}}(t) = \int_0^t u_{\mathrm{e}}(\tau) h_{\mathrm{e}}(t-\tau) d\tau + r_{\mathrm{e}}(0)(l_1 e^{\frac{-t}{T_1}} + l_2 e^{\frac{-t}{T_2}} + l_3 e^{\frac{-t}{T_3}}), \tag{3}$$

$$r_{\mathrm{h}}(t) = \int_0^t u_{\mathrm{h}}(\tau) h_{\mathrm{h}}(t-\tau) d\tau, \tag{4}$$

where $r_{\mathrm{e}}(0)$ is the initial eye position, and the initial head position is assumed to be zero. Other initial

conditions are $\dot{r}_{\mathrm{e}}(0) = \ddot{r}_{\mathrm{e}}(0) = 0$ and $\dot{r}_{\mathrm{h}}(0) = 0$. The coefficients l_1, l_2, and l_3 are defined as:

$$\begin{aligned} l_1 &= \frac{T_1^2}{T_1^2 + T_2T_3 - T_3T_1 - T_1T_2}, \\ l_2 &= \frac{T_2^2}{T_2^2 + T_3T_1 - T_1T_2 - T_2T_3}, \\ l_3 &= \frac{T_3^2}{T_3^2 + T_1T_2 - T_2T_3 - T_3T_1}, \end{aligned}$$

2 Gradient Descent Optimization

We use a gradient descent optimization method for minimizing the cost function. To this end, we start with calculating the partial derivative of the cost function with respect to each eye weight parameter:

$$\begin{aligned} \frac{\partial E}{\partial w_{ij}^{\mathrm{e}}} &= \int_0^T \frac{\partial}{\partial w_{ij}^{\mathrm{e}}} |r_{\mathrm{g}}(t)| dt + \alpha_{\mathrm{e}} \frac{\partial}{\partial w_{ij}^{\mathrm{e}}} \sum_{j=1}^{N} \sum_{i=1}^{M} (w_{ij}^{\mathrm{e}})^4 \\ &= -\int_0^T \frac{\partial |r_{\mathrm{g}}(t)|}{\partial r_{\mathrm{e}}(t)} \frac{\partial r_{\mathrm{e}}(t)}{\partial w_{ij}^{\mathrm{e}}} dt + 4\alpha_{\mathrm{e}} (w_{ij}^{\mathrm{e}})^3 \\ &= -\int_0^T \mathrm{sgn}(r_{\mathrm{g}}(t)) \frac{\partial r_{\mathrm{e}}(t)}{\partial w_{ij}^{\mathrm{e}}} dt + 4\alpha_{\mathrm{e}} (w_{ij}^{\mathrm{e}})^3, \end{aligned} \tag{5}$$

where the signum function, sgn(x), is defined as:

$$\mathrm{sgn}(x) = \begin{cases} -1 & \text{if} \quad x < 0; \\ 0 & \text{if} \quad x = 0; \\ +1 & \text{if} \quad x > 0. \end{cases}$$

Using Equation 4 in the paper we can calculate the partial derivative of $r_{\mathrm{e}}(t)$ with respect to w_{ij}^{e}:

$$\begin{aligned} \frac{\partial r_{\mathrm{e}}(t)}{\partial w_{ij}^{\mathrm{e}}} &= \frac{\partial}{\partial w_{ij}^{\mathrm{e}}} \int_0^t u_{\mathrm{e}}(\tau) h_{\mathrm{e}}(t-\tau) d\tau \\ &= \int_0^t \frac{\partial u_{\mathrm{e}}(\tau)}{\partial w_{ij}^{\mathrm{e}}} h_{\mathrm{e}}(t-\tau) d\tau, \end{aligned}$$

and since

$$\begin{aligned} \frac{\partial u_{\mathrm{e}}(t)}{\partial w_{ij}^{\mathrm{e}}} &= \frac{\partial}{\partial w_{ij}^{\mathrm{e}}} \sum_{j=1}^{N} \sum_{i=1}^{M} w_{ij}^{\mathrm{e}} s_{ij}(t) \\ &= s_{ij}(t), \end{aligned}$$

we obtain:

$$\frac{\partial r_{\mathrm{e}}(t)}{\partial w_{ij}^{\mathrm{e}}} = \int_0^t s_{ij}(\tau) h_{\mathrm{e}}(t-\tau) d\tau. \tag{6}$$

By substituting (6) into (5), the final form of the error function gradient is obtained:

$$\frac{\partial E}{\partial w_{ij}^{\mathrm{e}}} = -\int_0^T \mathrm{sgn}(r_{\mathrm{g}}(t)) \left(\int_0^t s_{ij}(\tau) h_{\mathrm{e}}(t-\tau) d\tau \right) dt + 4\alpha_{\mathrm{e}} (w_{ij}^{\mathrm{e}})^3. \tag{7}$$

Similarly, we can obtain the gradient with respect to the head weight parameters:

$$\frac{\partial E}{\partial w^{\mathrm{h}}_{ij}} = -\int_0^T \mathrm{sgn}(r_{\mathrm{g}}(t)) \left(\int_0^t s_{ij}(\tau) h_{\mathrm{h}}(t-\tau) d\tau \right) dt + 4\alpha_{\mathrm{h}} (w^{\mathrm{h}}_{ij})^3. \tag{8}$$

The weight adaptation rules are defined according to the gradient descent method as:

$$w^{\mathrm{e}}_{ij} \leftarrow w^{\mathrm{e}}_{ij} - \delta^{\mathrm{e}}_{ij} \frac{\partial E}{\partial w^{\mathrm{e}}_{ij}}, \tag{9}$$

$$w^{\mathrm{h}}_{ij} \leftarrow w^{\mathrm{h}}_{ij} - \delta^{\mathrm{h}}_{ij} \frac{\partial E}{\partial w^{\mathrm{h}}_{ij}}, \tag{10}$$

where δ^{e}_{ij} and δ^{h}_{ij} represent adaptation rates for the eye and head controller weights, respectively. These are adapted themselves as described in the next section.

3 Adaptive Learning Rate Method

For fast convergence with the gradient descent approach, we use an adaptive learning rate. The method we use is quite similar to the RPROP algorithm [6] with a slight modification: instead of using the sign of the error for updating the weights, we directly use the error value. This method provides a local adaptive learning scheme where the learning rate is adapted according to changes in the sign of the gradient. If the gradient has the same sign in two successive iterations, the learning rate of the corresponding weight parameter increases by a factor η^+; otherwise, it decreases by another factor η^-. This adaptation scheme can be formalized as:

$$\delta^{k+1}_{ij} = \begin{cases} \eta^+ \delta^k_{ij} & \text{if} \quad \frac{\partial E}{\partial w_{ij}}^k \cdot \frac{\partial E}{\partial w_{ij}}^{k+1} > 0, \\ \eta^- \delta^k_{ij} & \text{if} \quad \frac{\partial E}{\partial w_{ij}}^k \cdot \frac{\partial E}{\partial w_{ij}}^{k+1} < 0, \\ \delta^k_{ij} & \text{if} \quad \frac{\partial E}{\partial w_{ij}}^k \cdot \frac{\partial E}{\partial w_{ij}}^{k+1} = 0, \end{cases}$$

where $k+1$ represents the current and k represents the previous iteration number of the adaptation procedure. We set $\eta^+ = 1.01$ and $\eta^- = 0.95$ in our simulations.

4 Implementation

In order to choose the free parameters of our model (α_{e} and α_{h}), we use a genetic algorithm (GA) with a population of 20 chromosomes, each one containing specific values of the free parameters. A fitness value is defined for each chromosome as the sum of squared errors (SSE) at specific points between the simulated behavior when the chromosome's free parameter values are used, and experimentally observed data. The GA mutation process adds a random number taken from a zero-mean Gaussian distribution to each parameter value of a parent chromosome in order to create a child chromosome. This distribution shrinks by time, such that at each generation m its standard deviation is obtained as:

$$\sigma_{\mathrm{m}} = \sigma_{\mathrm{m-1}}(1 - \frac{mS}{N})$$

where N is the total number of generations and S controls how the standard deviation shrinks as the algorithm proceeds. We set $N = 48$ and $S = 0.15$ in our simulations. For each set of parameter values, the fitness value is calculated after the learning procedure (Equations 9 and 10) converges.

We used the C++ programming language to run the simulations, and MATLAB for illustration and statistical analysis. The simulations were carried out using a parallel computing implementation, such

that the chromosomes of a current generation of GA were simulated simultaneously. Furthermore, various instances of each chromosome pertaining to different gaze shift amplitudes were simulated in parallel. For more information about the genetic algorithm procedure used in this study, we refer the interested reader to [7].

References

1. Keller EL (1973) Accommodative vergence in the alert monkey. Motor unit analysis. Vision Res 13: 1565-1575.

2. Bizzi E, Dev P, Morasso P, Polit A (1978) Effect of load disturbances during centrally initiated movements. J Neurophysiol 41: 542-556.

3. Kalesnykas RP, Sparks DL (1996) The primate superior colliculus and the control of saccadic eye movements. Neuroscientist 2: 284-292.

4. Shadmehr R, Orban de Xivry JJ, Xu-Wilson M, Shih TY (2010) Temporal discounting of reward and the cost of time in motor control. J Neurosci 30: 10507-10516.

5. Freedman EG (2001) Interactions between eye and head control signals can account for movement kinematics. Biol Cybern 84: 453-462.

6. Riedmiller M, Braun H (1993) A direct adaptive method for faster backpropagation learning: The RPROP algorithm. In: IEEE International Conference on Neural Networks (ICNN). pp. 586-591.

7. Dorsey RE, Mayer WJ (1995) Genetic algorithms for estimation problems with multiple optima, nondifferentiability, and other irregular featues. J Bus Econ Stat 13: 53-66.
